# Supplementary material for: Content-rich biological network constructed by mining PubMed abstracts
Source: BMC Bioinformatics. 2004 Oct 8;5:147. doi: 10.1186/1471-2105-5-147 (PMC528731; doi:10.1186/1471-2105-5-147)
Supplement: Additional File 2 — The original results of the above study (non-essential files are deleted to keep the file size under the limit set by BMC bioinformatics). [file 1471-2105-5-147-S2.bz2 › chilibotAdditionalFile2/dip30/233ID10903799E757/html/IL2RB_IL2RG.html]

 


 **IL2RB** and **IL2RG** 
  
Found 102 abstracts in PubMed, retrieved 30.  
 

 What does Google say? 
 PDF only 
| .edu only 

---

**Interactive relationship** (e.g. stimulation, inhibition, etc)

**Stimulatory relationship**- Interleukin IL 2 receptor  [ **IL2RB** ]  subunit alpha IL 2Ralpha increases the affinity of the IL 2 receptor  [ **IL2RB** ]  complex while hetero association of IL 2Rbeta and gamma c  [ **IL2RG** ]  chains initiates a proliferative signal.  Ref: 11886175 Cytokine, 2002
**Neutral relationship**- One of these viral proteins, p12 I, encoded by HTLV I orfI, is a weak oncogene that binds the 16 kDa subunit of the vacuolar ATPase and interacts with the immature beta and gamma c  [ **IL2RG** ]  chains of the IL 2 receptor  [ **IL2RB** ] .  Ref: 10936091 Virology, 2000
- X linked severe combined immunodeficiency XSCID  [ **IL2RG** ]  is caused by mutations in the IL 2 receptor  [ **IL2RB** ]  gamma chain  **IL2RG**  gene, resulting in absent T lymphocytes and nonfunctional B lymphocytes.  Ref: 11319919 Mol Ther, 2001
- We have shown that retrovirus mediated transfer of the gamma c  [ **IL2RG** ]  cDNA induced gamma c  [ **IL2RG** ]  chain expression and restored the function of the high affinity IL 2 receptor  [ **IL2RB** ]  on SCI X1 EBV transformed B cell lines.  Ref: 11265225 Pathol Biol Paris , 2001

**Non-interactive relationship** (e.g. studied together, co-existance, homology, etc.)

- The immunophenotype of KHYG 1 cells was CD1, CD2, surface CD3, cytoplasmic CD3epsilon, CD7, CD8alphaalpha, CD16, CD25, CD33, CD34, CD56, CD57, CD122  [ **IL2RB** ] , CD132  [ **IL2RG** ] , and TdT.  Ref: 10803526 Leukemia, 2000
- Cell surface expression of CD25, CD122  [ **IL2RB** ] , CD132  [ **IL2RG** ] , and CD69 were also determined and no statistical differences were found between both groups.  Ref: 12635575 Allergy Asthma Proc< MedlineTA>Allergy Asthma Proc, 2000
- Both protein and mRNA for CD122  [ **IL2RB** ]  and CD132  [ **IL2RG** ]  were observed but there was no evidence of CD25 expression.  Ref: 11238664 J Immunol, 2001
- The expression of the IL 2R alpha, beta, and gamma chains, CD25, CD122  [ **IL2RB** ] , and CD132  [ **IL2RG** ] , respectively, was investigated on fibroblast like synoviocytes FLS and dermal fibroblasts DF.  Ref: 11238664 J Immunol, 2001
- This effect was found to involve down regulation of the expression of both CD25 IL 2R alpha and CD122  [ **IL2RB** ]  IL 2R beta on the lymphocyte membrane and a marked decrease in the level of up regulation of the expression of surface CD132  [ **IL2RG** ]  IL 2R gamma or gamma c  [ **IL2RG** ] .  Ref: 10359123 Eur J Immunol, 1999
- This was denoted by comparable mean fluorescence intensity values representing the expression of CD122  [ **IL2RB** ] , CD132  [ **IL2RG** ] , CD86 or CD80 molecules on the surface of SACI stimulated CD19 cells cultured without or with AGC10.  Ref: 11226453 Int J Parasitol, 2001
- This alteration was accompanied by others such as decreased expression of the CD122  [ **IL2RB** ]  and CD132  [ **IL2RG** ]  chains of the IL 2R complex.  Ref: 11226453 Int J Parasitol, 2001
- However, in marked contrast to LPS, the bacterial superantigen Staphylococcus aureus enterotoxin B SEB induced comparable serum levels of IFN gamma in IL 18 and IL 18 mice, accompanied by an upregulation of cell surface markers CD14, CD122  [ **IL2RB** ]  IL 2Rbeta, and CD132  [ **IL2RG** ]  IL 2Rgamma on peritoneal macrophages.  Ref: 10816504 Infect Immun, 2000
- No significant differences between cell lines were observed in IL 2 receptor  [ **IL2RB** ]  chain alpha, beta, or gamma c  [ **IL2RG** ]  expression, in IL 2 mediated proliferation, or in IL 2 induced phosphorylated forms of Stat3, Stat5, Jak1, or Jak3.  Ref: 10516077 J Virol, 1999
- Fairly large subpopulations of, but not all, B lymphocytes had their expression of CD122  [ **IL2RB** ] , CD132  [ **IL2RG** ] , CD86 and CD80 reduced to undetectable levels in the presence of AGC10.  Ref: 11226453 Int J Parasitol, 2001
- Supporting this view were the substantially diminished levels of IL 2, CD25, CD122  [ **IL2RB** ] , and CD132  [ **IL2RG** ]  mRNA found in AGC10 containing cultures of PHA activated lymphocytes.  Ref: 12467977 Mol Biochem Parasitol< MedlineTA>Mol Biochem Parasitol, 2001
- The beta c and gamma c  [ **IL2RG** ]  subunits of the IL 2 receptor  [ **IL2RB** ]  IL 2R are sufficient and necessary for signal transduction.  Ref: 11750878 Cytokine Growth Factor Rev, 2002
- Cells stained with Annexin V Anx and antibodies to CD56, CD3, CD95, CD25, CD122  [ **IL2RB** ]  or CD132  [ **IL2RG** ]  were examined by flow cytometry.  Ref: 12594840 Eur J Immunol, 2003
- Functional IL 2 receptor  [ **IL2RB** ]  beta CD122  [ **IL2RB** ]  and gamma CD132  [ **IL2RG** ]  chains are expressed by fibroblast like synoviocytes activation by IL 2 stimulates monocyte chemoattractant protein 1 production.  Ref: 11238664 J Immunol, 2001
- Engagement of interleukin 2 IL 2 mediates the heterodimeridation of the common beta chain beta c and common gamma chain gamma c  [ **IL2RG** ]  of the IL 2 receptor  [ **IL2RB** ]  IL 2R.  Ref: 10889465 Cell Signal, 2000
- Like living Trypanosoma cruzi, its AGC10 membrane glycoprotein inhibits interleukin 2 IL 2 secretion and membrane expression of CD25, CD122  [ **IL2RB** ] , and CD132  [ **IL2RG** ]  the components of the high affinity IL 2 receptor  [ **IL2RB** ]  by activated human lymphocytes.  Ref: 12467977 Mol Biochem Parasitol< MedlineTA>Mol Biochem Parasitol, 2000
- The receptors for interleukin 2 IL 2 and interleukin 15 IL 15 in T cells share the IL 2R beta subunit CD122  [ **IL2RB** ]  and gamma C  [ **IL2RG** ]  subunit but have private alpha subunits.  Ref: 12121224 Immunol Cell Biol, 2002
- The primary interleukin 4 IL 4 receptor complex on monocytes type I IL 4 receptor includes the 140 kDa alpha chain IL 4R alpha and the IL 2 receptor  [ **IL2RB** ]  gamma chain, gamma c  [ **IL2RG** ] , which heterodimerize for intracellular signaling, resulting in suppression of lipopolysaccharide LPS inducible inflammatory mediator production.  Ref: 10534111 J Leukoc Biol, 1999
- Janus kinase 3 JAK3 is a cytoplasmic tyrosine kinase required for T cell development and activated by cytokines that utilize the interleukin 2 IL 2 receptor  [ **IL2RB** ]  common gamma chain gamma c  [ **IL2RG** ] .  Ref: 10380915 J Leukoc Biol, 1999
- We have also characterized the genomic structure of the murine Tslpr gene which shows that in addition to primary sequence homology, it shares a common genomic organization of coding exons with the murine IL 2 receptor  [ **IL2RB** ]  common gamma chain  **Il2rg** .  Ref: 11891057 Gene, 2002
- AGC10 also decreased the intracellular levels of CD25, CD122  [ **IL2RB** ] , and CD132  [ **IL2RG** ]  in CD4 and CD8 cells stimulated with the T cell mitogen phytohemagglutinin PHA.  Ref: 12467977 Mol Biochem Parasitol< MedlineTA>Mol Biochem Parasitol, 2002
- A significant decrease of the basophilic surface antigens CD11c, CD32, CD35, CD63, CD116, CD122  [ **IL2RB** ] , CD124, CD130, and CD132  [ **IL2RG** ]  interleukin receptor gamma was detected 1 week after the end of rush VIT.  Ref: 11112905 J Allergy Clin Immunol, 2000
